# A Hypersexuality Subset Behavior Induced by Aripiprazole Overdose in an Antipsychotic Naïve Patient: A Case Report and Review of the Literature

**DOI:** 10.3390/clinpract16010019

**Published:** 2026-01-20

**Authors:** Tiziano Serfilippi, Silvia Piccirillo, Alessandra Preziuso, Valentina Terenzi, Francesca Romagnoli, Marella Tarini, Vincenzo Lariccia, Agnese Secondo, Simona Magi

**Affiliations:** 1 Department of Biomedical Sciences and Public Health, School of Medicine, University “Politecnica delle Marche”, Via Tronto 10/A, 60126 Ancona, Italy; t.serfilippi@pm.univpm.it (T.S.); s.piccirillo@staff.univpm.it (S.P.); a.preziuso@staff.univpm.it (A.P.); v.terenzi@staff.univpm.it (V.T.); v.lariccia@staff.univpm.it (V.L.); 2 Servizio Territoriale Dipendenze Patologiche, Ospedale di Senigallia, AST-Ancona, Via Cellini 1, 60019 Senigallia, Italy; francesca.romagnoli@sanita.marche.it (F.R.); marella.tarini@sanita.marche.it (M.T.)

**Keywords:** aripiprazole, hypersexuality, case report, dopamine, sexual, addiction

## Abstract

**Background**: Aripiprazole is an atypical antipsychotic that acts as a partial agonist on the dopamine receptor D2 while also displaying agonistic activity on the 5-HT1A and antagonistic activity on the 5-HT2A receptors. As a partial agonist, aripiprazole stabilizes the activity of the D2 receptor, preventing overactivation. **Case presentation**: Within our deprescribing activity, we came across the case of a 30-year-old antipsychotic-naïve patient treated with the depot formulation of aripiprazole for bipolar disorder and acute mania, possibly developing hypersexuality due to an overdose that impacted negatively and heavily on his personal life. **Results**: The patient developed a peculiar subset of hypersexuality, changing his sexual orientation. Of interest, one month after discontinuing aripiprazole and switching to paliperidone, all the sexual-related symptoms and impulse control disorders resolved. **Conclusions**: We suggest stronger communication among the clinical teams involved in the patient’s care and screening patients for impulse control disorder prior to the administration of aripiprazole and monitoring them during treatment.

## 1. Introduction

Aripiprazole is an atypical antipsychotic drug that belongs to the quinolone derivative family [1]. It binds more strongly to dopamine receptors (with a Ki of 0.74 nmol/L) than other antipsychotic drugs. Unlike first and other second-generation antipsychotics, aripiprazole acts as a partial agonist at the D2 receptor. This allows aripiprazole to stabilize the activity of the D2 receptor by hampering its hyperactivation in the striatal regions [1] and activating hypofunctional dopaminergic systems in the prefrontal cortex [2].

Aripiprazole has a complex activity on serotoninergic receptors, acting as a partial 5-HT1A agonist (with a Ki of 5.6 nmol/L) and a 5-HT2A antagonist (with a Ki of 35 nmol/L) [3,4,5,6].

Moreover, aripiprazole has a plethora of effects on many other receptors (namely α1A, H1, α1B, α2C, α2A, 5-HT1D), but such effects are marginal at clinically available concentrations [3].

Aripiprazole has been shown to be effective in treating both positive and negative symptoms of schizophrenia with a good safety profile [6], having a low incidence of side effects such as sleepiness, extrapyramidal symptoms, type II diabetes, short-term weight gain, movement disorders, and a reduction (instead of an elevation, commonly caused by other antipsychotics) in prolactin levels [1,4]. However, common side effects reported with aripiprazole include headache, agitation, insomnia, anxiety, constipation, dry mouth, orthostatic hypotension, and blurred vision [1]. Moreover, in 2016, it was found by the FDA that aripiprazole-induced impulse control disorders, including compulsive eating, shopping, and hypersexuality, significantly impaired patients’ daily lives [7].

## 2. Case Description

**Figure 1:**
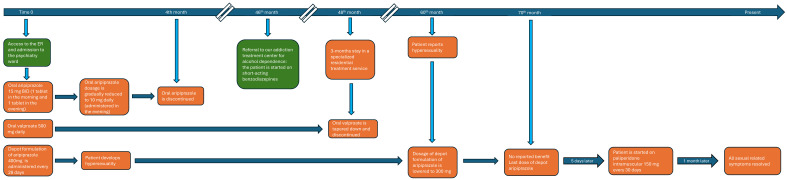
Timeline summarizing patient’s treatments.

A 30-year-old man was brought to the Emergency Room due to a severe manic episode and was later admitted to the psychiatric ward with the diagnosis of acute mania in bipolar disorder (Figure 1).

The patient was started on oral aripiprazole for 6 days. During this period, the dosage was initially 15 mg twice daily and was tapered down to 10 mg. After 6 days, aripiprazole 400 mg in depot formulation was administered via the intramuscular route every 28 days, while continuing oral aripiprazole 10 mg for four months, thereby configuring an overdose. Of note, before every administration of parental aripiprazole, a routine clinical follow-up visit was performed by the psychiatry team.

The patient was also started on slow-release valproate 500 mg once daily.

As a stable compensation of the patient’s mood and mental state was achieved in the following months, the oral formulation of aripiprazole was tapered down to 10 mg once daily and was eventually discontinued after approximately 4 months.

Shortly after receiving the first dose of aripiprazole, the patient developed hypersexuality and sexual disinhibition, which led him to unreasonable and dangerous sexual behaviors, which included requesting sex from his wife multiple times a day, cheating, performing unprotected sexual acts with prostitutes, excessive and compulsive masturbation, fixed and recurring thoughts on sex-related topics, porn addiction, and flirting even when not appropriate. Furthermore, the patient disclosed a change in sexual orientation. Prior to initiating depot aripiprazole, the patient reported being heterosexual. However, following the initiation of this treatment, the patient exhibited a pansexual orientation, with a significant focus on transgender individuals. Additionally, the patient reported the emergence of other impulse control disorders, including gambling and suicidal ideation.

Three years after his first admittance to the psychiatric ward, the patient was referred to our addiction treatment center for Alcohol Dependence. At this time, the patient was also diagnosed with Adjustment Disorder and had developed anxiety, which was initially treated with short-acting benzodiazepines. However, later on, the patient developed Benzodiazepine Dependence and Abuse.

Four years after his first admittance to the psychiatric ward, the slow-release valproate 500 mg once daily was tapered down and eventually discontinued during a 3-month stay in a residential treatment service specialized in addiction recovery.

Therefore, he was started on delorazepam (a long-acting benzodiazepine), and pregabalin and propranolol were added to the therapy regimen in order to treat his anxiety.

Approximately five years after being first started on aripiprazole, the patient reported compulsive symptomatology to the psychiatry team and addiction specialists and attributed it to aripiprazole. Therefore, aripiprazole was tapered down to 300 mg/fl/im once every 28 days, with a mild and temporary improvement in the reported symptomatology.

Eventually and recently, the patient’s sexually related symptoms took a heavy toll on his personal life and, after careful consideration of the cost–benefit ratio, the decision was made to switch from aripiprazole to a depot formulation of paliperidone 150 mg, administered once every 28 days.

The patient later reported having high libido since adolescence, which caused him to underestimate his symptoms and not report them for years; this, combined with the efficacy of aripiprazole in controlling his psychotic disorder and the clinicians not enquiring about it, led to the administration of aripiprazole 400 mg/fl/im once every 28 days for 5 years.

About one month after the last dose of the depot formulation of aripiprazole, all sexual-related symptoms, as well as all other impulse-control-related disorders and suicidal ideation reported by the patient, resolved.

For context, there were two teams involved in the management of our patient, the psychiatry team and the addiction specialist team, working in the same complex building.

Following the Naranjo Algorithm [8], hypersexuality, change in sexual orientation, and suicidal ideation are all likely caused by aripiprazole, as shown in Table 1.

Table 1 showing that hypersexuality and change in sexual orientation are likely caused by aripiprazole.

## 3. Discussion

The present case report discusses a peculiar subset of hypersexuality behavior in a 30-year-old antipsychotic-naïve patient consisting of a change in sexual orientation following the treatment with the depot formulation of aripiprazole for bipolar disorder and acute mania. Of note, he developed a pansexual orientation with a significant focus on transgender individuals, highly compromising his familial and social life.

The regulation of libido and sexual desire is extremely complex and is delegated to various structures of the central nervous system (CNS), such as the hypothalamus, amygdala, hippocampus, and nuclei of the septal region. Particularly, dopamine release in the nucleus accumbens is related to sexual reward [9] and motivation [10,11]. Therefore, it has been hypothesized that an upregulation of dopamine receptors or dopamine-dependent pathways may cause a shift towards hypersexuality [12]. Indeed, the use of dopamine agonists for Parkinson’s disease has been linked to the development of hypersexuality [13], while the use of dopamine antagonists has been associated with the onset of reduced libido and anorgasmia [14]. Of interest, it has been reported that adjunctive treatment of schizophrenia with aripiprazole improves sexual dysfunctions, such as erectile dysfunction, irregular menstrual cycle, and galactorrhea [15].

Still, it has been described how different dopamine agonists used to treat Parkinson’s disease, hyperprolactinemia, and restless leg syndrome may cause other impulse control disorders such as hypersexuality [13,16]. Of note, a study by Moole et al. found that the incidence of such adverse effects was higher with agonists that have a stronger affinity with the D3 receptor, but were still observed to a lesser extent with aripiprazole [16].

Additionally, Lawler et al. suggested that aripiprazole is not a partial agonist, but rather an agonist whose effects depend on the cellular location of the D2 receptor and the signaling pathways related to that receptor [17,18]. Therefore, aripiprazole-induced hypersexuality may be a result of the dopaminergic activity of aripiprazole on the nucleus accumbens and the whole mesolimbic circuit [5].

There is a growing number of case reports regarding the hypersexuality induced by aripiprazole, but usually in such reports patients have been previously treated with another antipsychotic medication [19,20,21,22]. This becomes relevant when we consider that receptor antagonists, according to the classical receptor theory, tend to induce a compensatory effect up-regulating receptor-modulated signaling responses. Hence, in said patients, the density of D2 receptors could have theoretically been increased by the previous use of typical antipsychotic agents, thereby leading to an increased responsiveness of the tissue to a following dopaminergic agonist exposure. Given that aripiprazole is a partial agonist, its agonistic profile could have prevailed in such conditions [5]. However, in the present case, the aforementioned mechanism can be excluded, since our patient did not assume any other antipsychotic agents before aripiprazole.

An explanation of the sexual behavior here reported may involve the activity of aripiprazole on the serotoninergic receptors [20]. Indeed, such activities (namely the partial agonism of 5-HT1A and the antagonism of 5-HT2A receptors) are similar to that of flibanserin, which is a 5-HT1A agonist and a 5-HT2A antagonist [4,23,24]. Flibanserin has been approved by the FDA in 2015 for the treatment of acquired, generalized hypoactive sexual desire disorder in premenopausal women [25]. Flibanserin (also referred to as the “female Viagra” or “pink Viagra”) has been surrounded by controversy from the very beginning, but it has shown a statistically significant improvement in one of the primary outcomes of the first two FDA trials, being the number of sexually satisfying events, and also a significant change in sexual desire, as reported in the sponsor’s follow-up study [23,26]. Given these statements, it is reasonable to assume that the agonism of 5-HT1A and the antagonism of 5-HT2A receptors can cause an increase in libido and sexual desire. Of interest, in a positron emission tomography study in humans, the effect in heterosexual men of the selective serotonin reuptake inhibitor (SSRI) fluoxetine were different from that of homosexual men [27]. Furthermore, Liu et al. [28] underlined the role for serotonergic signaling in mouse sexual preference. Therefore, it is possible to hypothesize that the hypersexuality and change in sexual orientation induced by aripiprazole may be well correlated with its serotoninergic activities.

Another hypothesis in our framework takes into account preclinical evidence showing that aripiprazole increases dopamine release in the prefrontal cortex of rats [29] and mice [30]. This brain region is extensively involved in the regulation of sexual behavior, likely through mechanisms involving 5-HT1A receptor activation and 5-HT2A receptor antagonism [31]. The resulting increase in dopamine levels is expected to activate multiple dopamine receptor subtypes, including D4 receptors.

The D4 receptors are considerably involved in the modulation of sexual behavior, since polymorphisms in their gene contribute to individual differences and orientation in sexual behavior in humans [32] and rodents [33]. Furthermore, polymorphisms in exon III of the human gene-encoding D4 receptor are associated with sexual promiscuity [34].

It is therefore reasonable to presume that dopamine release in the prefrontal cortex induced by aripiprazole may stimulate D4 receptors, in consideration of its moderate affinity for this subtype of receptors [18,35], thus contributing to the onset of hypersexual behavior characterized by promiscuity and a change in sexual orientation.

Accordingly, an interesting preclinical study suggested that excessive dopamine release can impact male sexual orientation in Drosophila, enhancing male–male courtship behavior [36].

Collectively, we suggest that aripiprazole be used with caution in patients with reported high libido, addictions, and other impulse control disorders related to pre-existing dopaminergic system dysregulation that could be secondarily exacerbated by the drug. Indeed, we should consider these conditions as risk factors for the development of aripiprazole-induced hypersexuality [19,22,37]. While aripiprazole is evidently capable of reaching a stable clinical compensation of mania, its increased prescription will obviously lead to the manifestation of rare and very rare side effects, such as impulse control disorders. Particular attention should be paid to the patients’ follow-up and the communication among clinical teams and psychosocial operators involved in the patients’ care.

Therefore, we must remain vigilant against drug overdosing and its potential side effects, revaluating our patients with a critical mindset.

## 4. Conclusions

Aripiprazole has a good safety profile, but it can provoke an increase in libido and obvious hypersexuality. Therefore, we suggest screening patients for impulse control disorder prior to the administration of aripiprazole and monitoring them during treatment for the emergence of hypersexuality and other impulse-control-related symptoms.

## Figures and Tables

**Table 1:** Naranjo Adverse Drug Reaction Probability Scale scores of the patients.

Adverse Event	Hypersexuality		Change in Sexual Orientation	
Question	Answer	Score	Answer	Score
Are there previous conclusive reports on this reaction?	Yes	+1	No	0
Did the adverse event occur after the suspected drug was administered?	Yes	+2	Yes	+2
Did the adverse reaction improve when the drug was discontinued or a specific antagonist was administered?	Yes	+1	Yes	+1
Did the adverse reaction reappear when the drug was readministered?	Do not know	0	Do not know	0
Are there alternative causes (other than the drug) that could have on their own caused the reaction?	No	+2	No	+2
Did the reaction reappear when a placebo was given?	Do not know	0	Do not know	0
Was the drug detected in the blood (or other fluids) in concentrations known to be toxic?	Do not know	0	Do not know	0
Was the reaction more severe when the dose was increased or less severe when the dose was decreased?	Yes	+1	Do not know	0
Did the patient have a similar reaction to the same or similar drugs in any previous exposure?	Do not know	0	Do not know	0
Was the adverse event confirmed by any objective evidence?	Yes	+1	No	0
Total score		8		5

## Data Availability

The data presented in this study are available on request from the corresponding authors due to its sensitive nature.
